# Trans-regulation and localization of orthologous maltose transporters in the interspecies lager yeast hybrid

**DOI:** 10.1093/femsyr/foy065

**Published:** 2018-06-19

**Authors:** Virve Vidgren, Brian Gibson

**Affiliations:** VTT Technical Research Centre of Finland Ltd, P.O. Box 1000, FI-02044 VTT, Finland

**Keywords:** Agt1, Malx1, α-glucoside transporters, brewer's yeast, *Saccharomyces eubayanus*, hybrid

## Abstract

In the interspecies lager yeast hybrid there are *MAL* loci involved in maltose and maltotriose utilization derived from each parent (*Saccharomyces cerevisiae* and *Saccharomyces eubayanus*). We show that trans-regulation across hybrid subgenomes occurs for *MAL* genes. However, gene expression is less efficient with non-native activators (trans-activation) compared to native activators (cis-activation). *MAL* genes were induced by maltose and repressed by glucose irrespective of host. Despite the strong expression of *S. cerevisiae*-type genes in the *S. eubayanus* host, a very low amount of transporter protein was actually observed in cells. This suggests that proper formation and configuration of the *S. cerevisiae* transporters is not efficient in *S. eubayanus*. The *S. eubayanus*-type Malx1 transporter was present in the plasma membrane in high amounts in all hosts (*S. cerevisiae*, *S. eubayanus* and *Saccharomyces pastorianus*) at all times. However, the *S. cerevisiae*-type transporters appeared sequentially in the plasma membrane; scMalx1 was localized in the plasma membrane during early to late linear growth and subsequently withdrawn to intracellular compartments. In contrast, the scAgt1 transporter was found in the plasma membrane mainly in the stationary phase of growth. Different localization patterns may explain why certain transporter orthologues in natural *S. pastorianus* strains were lost to mutation.

## Teaser

Gene transcription regulation across subgenomes of hybrid yeast.

## INTRODUCTION

Maltose and maltotriose comprise the majority of the fermentable sugar of wort, and utilization of these two sugars influences overall fermentation performance of brewer's yeast (Rautio and Londesborough 2003; Stambuk *et al.*^2006^). Genes for maltose and maltotriose transporters are located in *MAL* loci, each of which typically contains three genes, one coding for a transmembrane maltose/maltotriose transporter, a second for maltase and a third for the activator of the two other genes of the locus. The lager yeast *Saccharomyces pastorianus* is a natural hybrid of *Saccharomyces cerevisiae* and *Saccharomyces eubayanus* (Libkind *et al.*^2011^), and contains *MAL* loci from both parents. There is at least one complete *MAL* locus from the *S. eubayanus* parent found in lager yeast that contains all three genes (Nakao *et al.*^2009^; Baker *et al.*^2015^). Whereas from the *S. cerevisiae* parent, lager yeast has inherited several *MAL* loci which are located on different chromosomes, e.g. in the A15 lager yeast strain there are intact *MAL1*, *MAL2*, *MAL3* and *MAL4* loci (Vidgren, Ruohonen and Londesborough 2005; Magalhães *et al.*^2016^). It is not known if *S. cerevisiae*-type (sc-type) activators can activate *S. eubayanus*-type (se-type) *MAL* genes and vice versa. Maltose induction is controlled by the MAL-activator elements binding to MAL sites in the promoters of *MAL* genes, whereas glucose repression is mediated through Mig1 transcription factors which also bind to promoters of *MAL* genes (Levine, Tanouye and Michels 1992; Hu *et al.*^1995^). Mig1 and MAL-activators compete for binding sites at *MAL* loci, and it has been suggested that this competition determines the balance between repression and induction (Wang, Sirenko and Needleman 1997). It is suggested that the MAL-activators require intracellular maltose to obtain active conformation (Wang, Sirenko and Needleman 1997) but there exist also mutated MAL-activators that are active in the absence of maltose, e.g. the MAL activator in the CEN.PK2-1D laboratory strain (Rodicio and Zimmerman 1985).

Trans-activation influences the phenotype of hybrid organisms (Tirosh *et al.*^2009^). In the present study, cross-genome regulation was investigated in relation to *MAL* genes. Two orthologous gene pairs *AGT1* and *MALx1* were studied in the *S. eubayanus*, *S. cerevisiae* and lager yeast *S. pastorianus* backgrounds. In addition to measuring gene expression, transporter-encoding genes were tagged with either Gfp or mCherry fluorescent tags to assess the amount and localization of each transporter in the different hosts. In this way, the localization pattern of transporter proteins in foreign and native hosts could be compared. Also, the effects of foreign and native transporter expression on growth, sugar utilization and ethanol production of each host were studied. Objectives of the study were (1) to determine if MAL activators can operate across genome boundaries, (2) to study the amount and localization of Agt1 and Malx1 transporters in different host backgrounds and (3) to ascertain why certain orthologues gained loss-of-function mutations during lager yeast evolution. It is known from earlier studies that in the lager hybrid, one gene of each orthologous pair, *AGT1* and *MALx1*, is non-functional. In regard to the *AGT1* gene, the mutated version is sc*AGT1* (Vidgren, Ruohonen and Londesborough 2005) whereas for the *MALx1* gene pair the mutated form is the *seMALx1* (Nakao *et al.*^2009^; Baker *et al.*^2015^).

## MATERIALS AND METHODS

### Strains

The *S. eubayanus* type strain VTT-C12902, here called C902, was from the VTT Culture Collection, Finland; deposited as CBS12357 at CBS-KNAW Fungal Biodiversity Centre. Two industrial brewer's yeasts, the lager strain A-63015 and ale strain A-75060, here called A15 and A60, respectively, were also obtained from the VTT Culture Collection. The *S. cerevisiae* laboratory strain were CEN.PK2-1D (VW-1B) (MATα, leu2-3/112 ura3-52 trp1-289 his3Δ1 MAL2-8c SUC2) and FY834 (MATα, his3Δ200 ura3-52 leu2Δ1 lys2Δ202 trp1Δ63 GAL2+)

### Strain construction

Four different hosts were transformed with a plasmid bearing a single copy of either sc- or se-type *AGT1* or *MALx1* genes. Genes were under the control of their own promoters and tagged either with mCherry or Gfp fluorophores. Those genes of orthologous gene pairs that were functional in the lager hybrid were amplified by PCR from the A15 strain, while those genes that had gained loss-of-function mutations in lager yeast were amplified from the corresponding parent strain. Thus, functional sc*MALx1* and se*AGT1* were from the lager yeast A15, whereas sc*AGT1* was from the *S. cerevisiae* parent (A60 ale yeast was used here as the *S. cerevisiae* reference) and se*MALx1* from *S. eubayanus*. Table 1 describes plasmids constructed in the present study and shows the location of amplified transporter genes in the original hosts. It has been shown earlier for Malx1 and Agt1 transporters that transformants containing tagged and untagged forms of the same transporter exhibited similar patterns of maltose transport activity through different growth phases. This suggests that the specific catalytic activities of the transporters were little changed by tagging and that the tagged and non-tagged transporters were processed similarly during intracellular trafficking (unpublished data). In addition, Agt1 transporters tagged either with mCherry or Gfp tags were studied in different host backgrounds. The amount of fluorescent signal measured was proportional for the Gfp and mCherry tags (unpublished data).

**Table 1.** Plasmids constructed for the present study.

Plasmid short name	Plasmid composition	Origin of transporter	Location of transporter gene in original species
Pl_scMALx1-mCherry	YEplac195-scMALx1-mCherry-KanMX	*S. pastorianus* A15	In sequenced lager strain WS34/70 in sc-type Chr II contig208.1^a^
Pl_seMALx1-Gfp	YEplac195-seMALx1-Gfp-KanMX	*S. eubayanus* type strain CBS12357^T^	In sequenced *S. eubayanus* type strain CBS12357^T^ in Chr XVI^c^
Pl_scAGT1-mCherry	YEplac195-scAGT1-mCherry-KanMX	*S. cerevisiae* ale A60	In sequenced ale GSY2239 in Contig00198^b^; in A60 ale in Chr VII^d^
Pl_seAGT1-Gfp	YEplac195-seAGT1-Gfp-KanMX	*S. pastorianus* A15	In sequenced lager strain WS34/70 in se-type Chr VII^a^
Pl_empty plasmid	YEplac195-KanMX		

^a^Nakao *et al.*^2009^.

^b^U’ren *et al.*^2015^.

^c^Libkind *et al.*^2011^.

^d^Vidgren *et al.*^2005^.

Strong constitutive promoter constructs of *MAL* genes prepared earlier were used as a starting material for constructs in this study. These constructs contained PGK1prom-scMALx1-mCherry-PGK1term, PGK1prom-seAGT1-GFP-PGK1term, TDH3prom-seMALx1-GFP-TDH3term and TDH3prom-scAGT1-mCherry-TDH3term. The GFP used in the constructs was a variant of GFP called enhanced green fluorescence protein and was described in Pöggeler *et al.* (2003). Red fluorescent protein used was the mCherry variant described in Shaner *et al.* (2004). By using the earlier construct TDH3prom-seMALx1-GFP-TDH3term as a template, seMALx1-GFP-TDH3term fragment was obtained by PCR. The seMALx1 promoter fragment (840 bp) was synthesized by PCR using *S. eubayanus* as a template. Each fragment was provided with short overlapping ends to help homologous recombination. Primers used for PCR reactions are listed in Table 2. Fragments generated were linked to each other and to the YEplac195 multicopy plasmid by homologous recombination near the SphI site previously cut with this enzyme. Yeast strain FY834 was used as a host in all recombination cloning steps of the present study similarly as in Vidgren *et al.* (2011). The PGK1prom-scMALx1-mCherry-PGK1 construct was used as a template to obtain a scMALx1-mCherry-PGK1 fragment. The scMALx1 promoter fragment (899 bp) was synthesized by PCR using A15 as a template. Fragments generated were linked to each other and to the YEplac195 plasmid by homologous recombination near the HindIII site previously cut with this enzyme. The TDH3prom-scAGT1-mCherry-TDH3term construct was used as a template to obtain scAGT1-mCherry-TDH3term fragment. The scAGT1 promoter fragment (1899 bp) was synthesized by PCR using A60 as a template. Fragments generated were linked to each other and to the YEplac195 plasmid containing an ura marker by homologous recombination near the SalI site previously cut with this enzyme. Similarly, the seAGT1-GFP-PGK1term fragment was obtained by using the earlier PGK1prom-seAGT1-GFP-PGK1t construct as a template. The seAGT1 promoter fragment (1898 bp) was synthesized by PCR using A15 as a template. Fragments generated were linked to each other and to the YEplac195 plasmid by homologous recombination near the HindIII site previously cut with this enzyme. All fragments were synthesized by PCR using standard methods and all homologous recombination reactions were performed as described in Vidgren *et al.* (2011). After verification of the sequences of each construct, transformation of the CEN.PK2-1D strain was performed using the lithium acetate transformation protocol (Gietz *et al.*^1992^). Transformants were identified using uracil selection. For growth in rich medium under G418 selection, a KanMX cassette which contains the kanamycin marker gene and confers resistance to the antibiotic G418 (Wach *et al.*^1994^) was used. The cassette was introduced to the seAGT1-GFP-Yeplac195 and scAGT1-mCherry-YEplac195 constructs as a NarI-XbaI fragment, to the scMALx1-mCherry-YEplac195 construct as SacI-XbaI fragment and to the seMALx1-GFP-YEplac195 construct as SalI-EcoRI fragment. The constructs obtained were transformed to the C902, A15 and A60 hosts using the lithium acetate transformation protocol, and transformants were selected using G418 (200 mg l^−^^1^). Table 3 describes all strains used in the present study.

**Table 2.** PCR primers used in the homologous recombination cloning.

Name^a^	Primer sequence	Sequence detected^b^
seAGT1prom_F	5΄-GCTATGACCATGATTACGCCAGAATTTAAAGGCGCGCGT -3΄	YEplac195 flanking sequence 5΄ of HindIII underlined, −1900 to −1878 of se*AGT1* promoter
seAGT1promR	5΄- AGCGAAAGTATATTTTTCATATTCTGATATTTCGTAACTCTTTCTGATTA -3΄	se*AGT1* gene 1–20 underlined, seAGT1 promoter from −1 to −30
seAGT1_F	5΄- TATCAGAATATGAAAAATATACTTTCGCTGGTAGGAAG -3΄	SeAGT1 prom from −1 to −10 underlined, seAGT1 gene from 1 to 29
PGKterm_R	5΄-TAGAGTCGACCTGCAGGC -’3΄	YEplac195 flanking sequence 3΄ of HindIII
scMALx1prom_F	5΄- GCTATGACCATGATTACGCC ATCAGAAATAGTCATTTATGTATTTTAGTTACGCT -3΄	YEplac195 flanking sequence 5΄ of HindIII underlined, −800 to −765 of se*MALx1* promoter
scMALx1_prom_R	5΄- ATGAGGATAATCCCTTCAT AGTTAATTAATAGTCTTGGATGTAATTCTTATTGT -3΄	sc*MALx1* gene underlined, scMALx1prom from −1 to −36
scMALx1-mCherry-PGKterm_F	5΄- TTAATTAACT ATGAAGGGATTATCCTCAT TAATAAACAGA -3΄	scMALx1 promoter from −1 to −10, scMALx1 gene
scAGT1prom_F	5΄- GATCCTCTAGAGTCGAGTTCTAGAAGCCTCCGGCAAA -3΄	YEplac195 flanking sequence underlined (SalI site), scAGT1prom
scAGT1_prom_R	5΄- CAATGAAATGATATTTTTCATATTATAATATTTTTTTAGTT GTTTGATGTTCTTCTATGTA -3΄	scAGT1 gene underlined from 1 to 21,scAGT1prom from −1 to −38
scAGT1_F	5΄- CAACTAAAAAAATATTATAATATGAAAAATATCATTTCATTGGTAAGCAAG -3΄	scAGT1prom from −1 to −21, scAGT1 gene from 1 to 30
TDH3_R	5΄-AAAGCTTGCATGCCTGCAGGTCGACATGTAGATGATACTGACTGCACG-3΄	Yeplac195 flanking sequence underlined (SalI), *TDH3* term
seMALx1_prom_F	5΄-CCTCTAGAGTCGACCTGCAGG TTCTGTACGCGATTTGGTTATGTG-3΄	YEplac195 flanking sequence underlined (SphI site), seMALx1prom
seMALx1_prom_R	5΄-CATTGAGGATAGACCCTTCATAGTTGCCTAATAGCTTTTGAAATTTTTT-3΄	*seMALx1* gene underlined from 1 to 21, seMALx1 promoter from −1 to −28
seMALx1_F	5΄-AAATTTCAAAAGCTATTAGGCAACTATGAAGGGTCTATCCTCAATGATAAAT-3΄	seMALx1 prom underlined from −1 to −25, seMALx1 from 1 to 27
seMALx1_R	5΄-AAATTCTGCGTTCGTTAAAGCTTG CATGTAGATGATACTGACTGCACG-3΄	YEplac195 flanking sequence underlined (SphI site), TDH3 terminator
TDH3prom_FRW(SalI)	5΄-ACCCGGGGATCCTCTAGAGTCGA CACGCTTTTTCAGTTCGAGT-3΄	Yeplac195 flanking SalI sequence underlined, TDH3promoter sequence
TDH3promREV_AGT1	5΄-AATGAAATGATATTTTTCATTTTGTTTGTTTATGTGTGTTTATTCGA-3΄	scAGT1 sequence underlined from 1 to 20, TDH3 prom
ScAGT1mCherry_FRW_TDH3p	5΄-TAAACAAACAAAATGAAAAATATCATTTCATTGGTAAG-3΄	TDH3 prom underlined, scAGT1 from 1 to 26
ScAGT1mCherry_REV_TDH3t	5΄-AAGATTTAAAGTAAATTCACTACAGCTCGTCCATGCC -3΄	TDH3term underlined, mCherry
TDH3term_FRW_mCherry	5΄-ATGGACGAGCTGTAGTGAATTTACTTTAAATCTTGCATTTAAAT-3΄	mCherry underlined, TDH3 term
TDH3term_REV_plasmidi (SalI site)	5΄- AAAGCTTGCATGCCTGCAGG CATGTAGATGATACTGACTGCACG-3΄	Plasmid 3΄ SalI site underlined, TDH3 term
TDH3p_FRW_SphI_site	5΄-CCTCTAGAGTCGACCTGCAGGCACGCTTTTTCAGTTCGAGT -3΄	Plasmid 5΄ SphI site underlined, TDH3 prom
TDH3p_REV_SeMALx1	5΄-TAGACCCTTCATTTTGTTTGTTTATGTGTGTTTATTCGA-3΄	seMALx1, TDH3prom
SeMALx1_FRW_TDH3prom	5΄-TAAACAAACAAA ATGAAGGGTCTATCCTCAATG-3΄	TDH3prom underlined, seMALx1
SbMALx1_REV_GFP	5΄-CGCCGCACTAGTTCATCTAATCATGAGAGATGGG-3΄	linker underlined, seMALx1
GFP_FRW(linker_SbMALx1)	5΄-TGAACTAGTGCGGCGATGGTGAGCAAGGGCGA-3΄	linker_seMALx1, GFP
GFP_REV (TDH3term)	5΄-AAGTAAATTCACTTACTTGTACAGCTCGTCCATGC-3΄	TDH3term, GFP
TDH3Term-FRW(Gfp)	5΄-CTGTACAAGTAA GTGAATTTACTTTAAATCTTGCATTTAAAT -3΄	GFP underlined, TDH3 term
TDH3term-REV(plasm)	5΄- TCTGCGTTCGTTAAAGCTT GCATGTAGATGATACTGACTGCACG-3΄	Yeplac195 flanking sequence underlined (SphI), TDH3 term

^a^F, forward; R, reverse.

^b^The numbering is from the first nucleotide of the translational start.

**Table 3.** Strains used in the present study.

Host	Species	Transformed plasmid
CEN.PK2-1D	*S. cerevisiae*	Pl_scMALx1-mCherry
CEN.PK2-1D		Pl_seMALx1-Gfp
CEN.PK2-1D		pl_scAGT1-mCherry
CEN.PK2-1D		pl_seAGT1-Gfp
CEN.PK2-1D		pl_empty plasmid
A60 ale	*S. cerevisiae*	Pl_scMALx1-mCherry
A60 ale		Pl_seMALx1-Gfp
A60 ale		pl_scAGT1-mCherry
A60 ale		pl_empty plasmid
C902	*S. eubayanus*	Pl_scMALx1-mCherry
C902		Pl_seMALx1-Gfp
C902		pl_scAGT1-mCherry
C902		pl_empty plasmid
A15 lager	*S. pastorianus*	Pl_scMALx1-mCherry
A15 lager		Pl_seMALx1-Gfp
A15 lager		pl_scAGT1-mCherry
A15 lager		pl_empty plasmid

### Cultivations

Native, non-laboratory yeasts bearing the constructs were grown in YP (10 g yeast extract and 20 g peptone l^−^^1^) containing 20 g l^−^^1^ maltose or glucose supplemented with G418 (200 mg l^−^^1^) at 24°C with 200 rpm shaking. Strains of the laboratory yeast CEN.PK2-1D bearing the constructs were grown in synthetic complete medium (Sherman, Fink and Hicks 1983) lacking uracil (SC-Ura) and containing either 20 g l^−^^1^ glucose or maltose at 30°C with 200 rpm shaking. Host strains transformed with empty plasmids were used as controls. Samples were collected from cultivations for OD_600_ determination, HPLC, RNA extraction and confocal microscopic studies.

### Total RNA extraction

Frozen cells were mechanically disrupted using a Precellys24 homogenizer (Bertin Technologies). Total RNA was extracted using the RNeasy mini kit (Qiagen). To eliminate genomic DNA, an additional DNase treatment was performed with RNase-free DNaseI (Thermo scientific) according to instructions. The extracted RNA was quantified using the ND-1000 UV-visible light spectrophotometer (NanoDrop Technologies). As another preliminary quality control assay, the absence of contaminant genomic DNA in RNA preparations was verified using RNA as a template in real-time PCR assays (RNA not reverse-transcribed to cDNA).

### Quantitative RT-qPCR

One microgram of total RNA from each sample was reverse transcribed to cDNA with the Transcriptor First Strand cDNA Synthesis kit (Roche) according to kit's instructions. A total of 5 μl of cDNA (1:100 diluted) was PCR amplified from each of the above reverse transcription reactions.

Primers were designed for specific binding to GFP, mCherry and the reference gene *IPP1* (Table 4) with the PerlPrimer v 1.1.21 program. The PCR efficiency of each primer pair (E) was evaluated by the dilution series method using a mix of sample cDNAs as a template. The efficiencies (E) of the qPCR assays (ranging from 1.94 to 2.00) for each primer pair were calculated using the formula 10^(^^−^^1/^m^)^, where m is the slope of the line of the threshold cycle (CT)-versus-log dilution plot of the DNA template (5 pg–50 ng input DNA) (Pfaffl 2001). Amplification efficiencies were for mCherry primer pair 94%, for Gfp 98% and for *IPP1* pair 96%. The PCRs were performed using a LightCycler^®^ 480 SYBR Green I Master mix (Roche Diagnostics) on a LightCycler^®^ 480 II instrument (Roche Diagnostics, Switzerland) in two independent replicates. The following program was used: pre-incubation (95°C for 5 min), amplification cycle repeated 45 times (95°C for 10 s, 60°C for 10 s, 72°C for 10 s with a single fluorescence measurement), melting curve program (65–97°C with continuous fluorescence measurement), and finally a cooling step to 40°C. Data analysis was performed using the supplied LightCycler^®^ 480 Software, version 1.5 (Roche Diagnostics) by the 2^−^^ΔΔCT^ method.

**Table 4.** Primers for RT-qPCR.

Name	Primer sequence	Sequence detected
mCherry_F	5΄-GCGTGATGAACTTCGAGGAC-3΄	299–318 of mCherry gene
mCherry_R	5΄-TTCAGCCTCTGCTTGATCTC-3΄	512–493 of mCherry gene
GFP_F	5΄-CACCATCTTCTTCAAGGACGA-3΄	291–311 of GFP gene
GFP_R	5΄-GGCTGTTGTAGTTGTACTCC-3΄	445–426 of GFP gene
IPP1_F	5΄-TTGCTTACACTGGTCAAGTC-3΄	385–405 of IPP1 gene
IPP1_R	5΄-AACCATTCGTTAGTAGCCCTC-3΄	549–579 of IPP1 gene

### Confocal microscopic studies

Cells containing GFP- or mCherry-tagged transporters were examined on Ibidi Ibitreat μ-Slides (Integrated Biodiagnostics). Confocal images were taken using a Zeiss Axiovert 200M with a spinning disc confocal unit Yokogawa CSU22 and Zeiss Plan-Neofluar 100xOil/1.4 NA objective. Image analysis was performed with SlideBook 4.2.0.7 software and cell-associated fluorescence was quantified using the National Institutes of Health (NIH) ImageJ program (http://rsbweb.nih.gov/ij/). A possible disadvantage of this method is that kinetics of the degradation of reporter proteins might be slower compared to transporter proteins. There is the possibility that fluorescent signal might be observed in cells when the transporters have already been degraded. The average fluorescence was calculated as total cell-associated signal intensity in 150–200 cells. Samples were collected for confocal microscopic analysis at the following time points, early linear phase (OD_600_ 2-4), late linear phase (OD_600_ 15-20) and stationary phase (after 40 h cultivation; OD_600_ >20) for native yeasts grown in rich media. For laboratory yeast grown in minimal media, samples were taken at the early linear phase (OD_600_ 2-4), late linear phase (OD_600_ 7-10) and stationary phase (after 40 h cultivation; OD_600_ >10).

### HPLC

The culture supernatants were analyzed by HPLC for maltose, glucose and ethanol using a Waters 2690 Separation Module and Waters System Interphase Module liquid chromatography coupled with a Waters 2414 differential refractometer and a Waters 2487 dual λ absorbance detector (Waters, Milford, MA). A Fast Juice Column (50 × 7.8 mm, Phenomenex, Torrance, CA) and a Fast Acid Analysis Column (100  × 7.8 mm, Bio-Rad, Hercules, CA) and an Aminex HPX-87H Organic Acid Analysis Column (300  × 7.8 mm, Bio-Rad) were equilibrated with 2.5 mM H_2_SO_4_ in water at 60°C and samples were eluted with 2.5 mM H_2_SO_4_ in water at a 0.5 ml/min flow rate. Data were acquired with Waters Millennium software.

## RESULTS

### Sequence comparisons

The gene encoding the se*-*type MAL activator is located in Chr XVI in *S. eubayanus* type strain CBS12357^T^ genome (Libkind *et al.*^2011^). This is the same locus from which the se*MALx1* transporter used in the present study as a transporter was amplified. There are no other MAL activator genes found in the genome of the sequenced *S. eubayanus* type strain. The sc-type MAL activator used in the sequence comparison is encoded by a gene located in contig208.1 of the sequenced WS34/70 lager yeast (Nakao *et al.*^2009^) in the same locus from which sc*MALx1* used in the present study as a transporter was obtained. In the lager WS34/70 genome there are three other sc-type MAL activators present and they have 95%, 97% and 98% sequence identities to the contig 208.1 activator.

**Figure 1.**
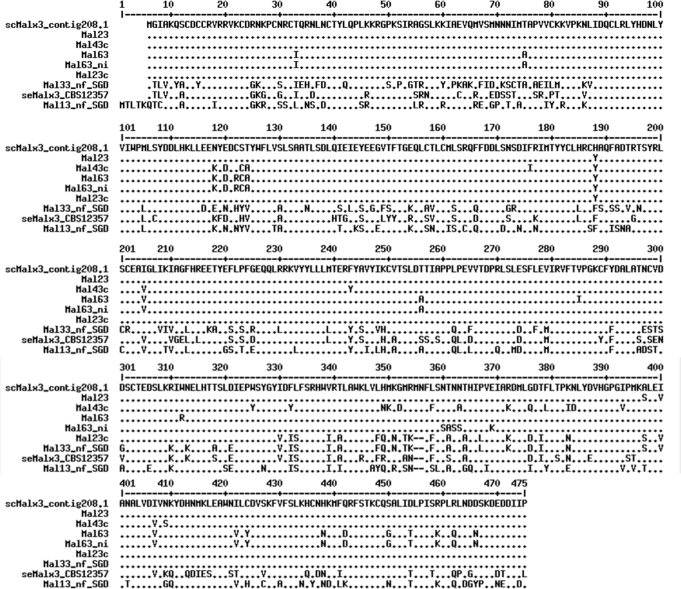
Comparison of the deduced amino acid sequences of se and sc-type MAL activators. Se-type MAL activator is encoded by *MALx3* located in Chr XVI in *S. eubayanus* type strain CBS12357^T^ (Libkind *et al.*^2011^). Sc-type MAL activator encoded by sc*MALx1* is present in the same locus as transporter gene used at the present study and is located in Chr II in WS34/70 (Contig 208.1) (Nakao *et al.*^2009^). In addition, Mal13 non-functional activator (accession number 853205), Mal23 activator (accession number AF002704), Mal23 constitutive activator (accession number AF002703) and Mal33 non-functional activator (accession number 852600). Mal43c constitutive activator (accession number M81157), Mal63 activator (accession number M6537) and Mal63 non-inducible activator (accession number AF003003) are included in the comparison. Multiple sequence alignment with hierarchical clustering (Corpet 1988).

A comparison of the *S. eubayanus* and *S. cerevisiae* MAL activators is shown in Fig. 1. Sequence identity of se-type activator to different sc-type activators varied from 69% to 74% at the protein level (Fig. 1). Regulatory domains of the MAL activator have been studied. It has been reported that the sequence-specific DNA binding domain of the MAL activator is contained in residues 1-100, residues 60-283 constitute a functional core region, including the transactivation domain, residues 251-299 are required to inhibit the activation function of MAL activator and the rest of the C-terminal region of the protein contains maltose responsive domains that act to relieve the inhibitory effect on the activation domain (Levine, Tanouye and Michels 1992; Hu *et al.*^1999^). Our comparison of se and sc-type MAL activators showed that changes are distributed evenly along the protein sequence and they are not concentrated in specific functional domains.

A phylogeny of the MAL activators shows three distinct subgroups of regulators (Brown, Murray and Verstrepen 2010). These are MAL63-like subgroup responding to several sugars (including maltose, maltotriose and palatinose), MAL13-like subgroup which is responsible only for palatinose; non-functional Mal13 and Mal33 activators of S288c belong to this group, even if they are ‘non-functional’ for maltose they respond to palatinose (Brown, Murray and Verstrepen 2010). A third subgroup contains putative maltose-responsive transcription factors whose sequences are not close to activators of the present study and for this reason were excluded from the sequence comparison shown in Fig. 1. Sequence analysis shows that se-type MAL activator and MAL13 subgroup activators (Mal13 and Mal33) share several of the amino acid changes in relation to MAL63-like subgroup. Thus, the seMALx1 activator is closer to Mal13-like subgroup than MAL63-like subgroup and due to this might be more responsive to palatinose than maltose.

Pougach *et al.* (2014) have studied amino acid changes in MAL activators responsible for the maltose to palatinose change, i.e. altered DNA-binding ability from CGC(9N)CGN to CGG(9N)CGG. They have identified Arg12Cys and Val13Ile amino acid changes to be responsible for binding ability change. However, there must be also other not yet identified changes as only Val13Ile change can be found in MAL13 subgroup promoters of the present study responding only to palatinose (in Mal13 activator). MAL63-type activators are promiscuous and can bind both CGC and CGG motifs (respond to both maltose and palatinose). The se-type MAL activator does not possess Arg12Cys and Val13Ile changes and, thus, there is so far no evidence that the se-type MAL activator is not a promiscuous activator able to bind both CGC and CGG motifs.

Relevant promoters se*MALx1*prom, sc*MALx1*prom, se*AGT1*prom and sc*AGT1*prom, were analyzed for elements responsible for MAL-activator and Mig1 repressor binding. Figure S1 (Supporting Information) shows sequence comparison of *MALx1* promoters and the divergent *MALx1-MALx2* promoter studied earlier (Levine, Tanouye and Michels 1992; Bell *et al.*^1997^), where two Mig1 binding sites and three MAL activator binding sites have been found. Table 5 shows sequence identities in promoter elements between sc and se-type promoters, i.e. MAL activator binding sites were 56%–84% identical whereas Mig1 repressor binding sites were only 56%–58% identical. *AGT1* promoters differentiated even more between sc and se-type (Table 6; Fig. S2, Supporting Information). MAL activator and Mig1 binding sites were only 33%–53% and 33%–58% identical, respectively. Sequence analysis indicated that trans-regulation of transporters would be unlikely, especially with the *AGT1* gene. However, based on current knowledge three-nucleotide CG-rich motifs separated by a spacer of fixed length are sufficient for a MAL activator to bind Pougach *et al.* (2014). Binding sites described in Pougach *et al.* were found in promoters in the present study. Figure S1 (Supporting Information) includes CGC(9N)CGN and CGG(9N)CGG binding sites in *MALx1* promoters and Fig. S2 (Supporting Information) those of *AGT1* promoters in addition to other described MAL activator binding sites described in Levine, Tanouye and Michels (1992), Bell *et al.* (1997) and Vidgren *et al.* (2011). There were two CGC(9N)CGN and CGG(9N)CGG binding sites in each of the sc and se type *MALx1* promoters. There were also two such sites in the se*AGT1* promoter and one site in A15 and A60 *AGT1* promoters.

**Table 5.** *MALx1* promoter element sequence identities between sc and se-type promoters. Location of elements in aligned sequences (Fig. S1, Supporting Information) is shown in parenthesis.

Element	se-type promoter element identity to sc-type (identical nucleotides/total nucleotides in element)	% Identity
Mig1 (283–300)	11/19	58
MAL activator (465–496)	27/32	84
MAL activator (542–559)	14/19	74
MAL activator (574–598)	14/25	56
Mig1 (599–614)	9/16	56

**Table 6.** *AGT1* promoter element sequence identities between sc and se-type promoters. Location of element in aligned sequences (Fig. S2, Supporting Information) is shown in parenthesis.

Element	se-type promoter element identity to sc-type (identical nucleotides/total nucleotides in element)	% Identity
MAL activator (812–827)	5/15	33
MAL activator (907–921)	4/15	27
MAL activator (943–957)	5/14	36
Mig1 (1088–1099)	4/12	33
Mig1 (1098–1110)	7/12	58
Mig1 (1109–1120)	7/12	58
Mig1 (1119–1130)	7/12	58
Mig1 (1128–1140)	6/12	50
Mig1 (1184–1195)	5/12	42
Mig1 (1195–1205)	5/12	42
MAL activator (1703–1717)	8/15	53
MAL activator (1882–1895)	6/14	43

### Gene transcription in the laboratory yeast CEN.PK2-1D

**Figure 2.**
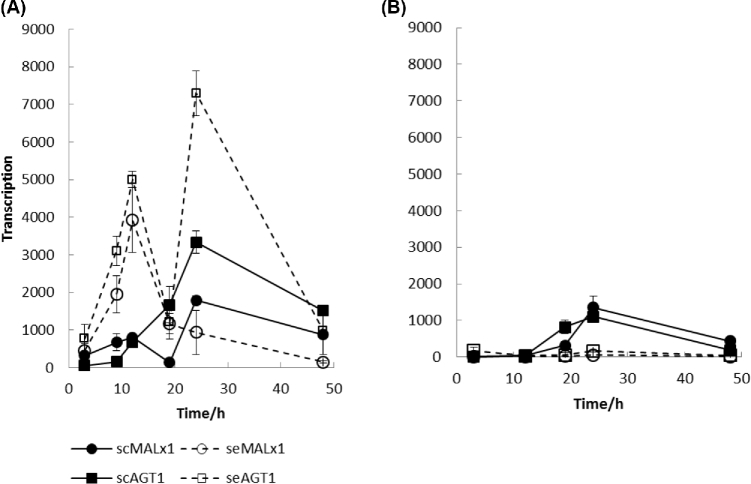
Gene transcription in the CEN.PK2-1D host transformed with sc and se-type transporters and grown on **(A)** maltose and **(B)** glucose. Results are averages ± ranges of data from two replicate growths of each yeast strain on each sugar.

Initially, transporter constructs were studied in the laboratory yeast CEN.PK2-1D containing a strong sc-type MAL activator in a MAL2-8c locus (Rodicio and Zimmermann 1985). Under inducing conditions (on maltose), the MAL activator present in CEN.PK2-1D was able to induce both sc- and se-type genes (both *MALx1* and *AGT1*). However, the se-type genes were transcribed at levels up to 10-fold higher than those of the sc-type genes (Fig. 2). There were two peaks in expression of the sc*MAL* genes in the CEN.PK2-1D host. The first peak was at early linear growth phase, when transcription of both se-type genes peaked, while transcription of sc-type genes remained at a low level. A second peak took place at late linear growth phase when both sc-type genes were induced as well as se*AGT1*, but not se*MALx1*. Under repressive conditions (on glucose) repression was very strong with se-type gene transcription being very weak, even after glucose exhaustion. In contrast, sc-type genes were transcribed relatively strongly after glucose exhaustion.

**Figure 3.**
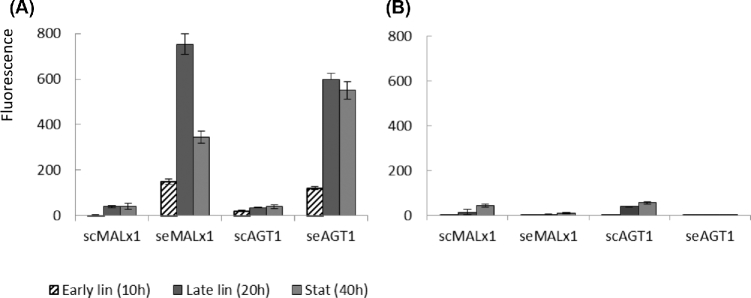
Amount of fluorescent signal in the CEN.PK2-1D host transformed with sc and se-type transporters and grown on **(A)** maltose and **(B)** glucose. Samples were collected at early logarithmic growth phase at 10 h, at late logarithmic growth phase at 20 h and at stationary growth phase at 40 h. Results are averages ± ranges of data from two replicate growths of each yeast strain on each sugar.

Next, the amount of fluorescence from each transporter was determined in cells. This was done by confocal microscopic analysis and signal quantification of mCherry or Gfp tagged transporters. The amount of tagged transporter (fluorescence) was determined for whole cells and thus included transporters present on the plasma membrane as well as in the intracellular compartments (Fig. 3). On maltose, the amount of fluorescence for both se-type transporters in cells was relatively high. For example, at stationary growth phase the se-type transporter fluorescence was 9–15 times higher than the corresponding fluorescence of sc-type transporters. Under repressive conditions (on glucose) there was a similar amount of fluorescence for sc-type transporter proteins as when grown under inducing conditions (on maltose), correlating to the observed transcription levels in both conditions; se-type transporter fluorescence was present at a very low basal level under repressive conditions, again correlating with transcription.

The division of transporters between plasma membrane and intracellular compartments was also studied by confocal microscopy. Figure 3 shows average total fluorescence in cells but it did not differentiate between fluorescence in the plasma membrane and intracellular compartments. In Table 7 fluorescence detected either in plasma membrane or in intracellular compartments, determined by visual inspection of fluorescent images, is shown. Different localization patterns were observed depending on transporter type. Under inducing conditions, seMalx1 transporter fluorescence was found consistently in the plasma membrane as well as in intracellular compartments during each growth phase. The sc-type version, scMalx1, was not present, or was below detection limit on the plasma membrane, but found only in intracellular compartments. For Agt1 transporters, a similar localization pattern was found for both the sc and se-type, with transporters in the plasma membrane from late linear until stationary phase. Under repressive conditions there were faint signals for scAgt1 and scMalx1 transporters in the plasma membrane and in intracellular compartments. The scMalx1 transporter signals were found in the plasma membrane only during late linear growth phase, while the scAgt1 transporter signals were found in the plasma membrane only in the stationary phase.

**Table 7.** Localization of tagged transporters in the CEN.PK2-1D host when grown on maltose or glucose in minimal media. Localization determined at early linear phase (OD_600_ 2-4), late linear phase (OD_600_ 7-10) and stationary phase (after 40 h cultivation; OD_600_ >10). Determined from fluorescent images by visual inspection.

On maltose	On plasma membrane			In intracellular compartments		
	Early lin	Late lin	Stat	Early lin	Late lin	Stat
scMalx1	−	−	−	+	+	+
seMalx1	++	++	++	++	++	++
scAgt1	−	+	+	+	+	+
seAgt1	−	++	++	++	++	++
	Early lin	Late lin	Stat	Early lin	Late lin	Stat
scMalx1	−	+	−	−	+	−
seMalx1	−	−	−	−	−	−
scAgt1	−	−	+	−	+	+
seAgt1	−	-	-	−	-	-

^−^ Not visible.

^+^ Fluorescent signals.

^++^ Strong fluorescent signals.

On maltose, contrary to what was expected, for strains possessing transporter constructs, utilization of maltose was not improved, but was rather slower (Fig. S3, Supporting Information). In particular, the scMALx1 construct caused a significant delay in growth, maltose utilization and ethanol production. On glucose, strains possessing a transporter construct had in many cases slightly slower glucose utilization capacity.

### Gene transcription in non-laboratory yeasts

Constructs were studied in native, non-laboratory yeast hosts, *S. cerevisiae* A60, *S. eubayanus* C902 and *S. pastorianus* A15 (hybrid of the first two species). In this manner, transregulation across genomes could be studied. Yeasts were transformed with the same sc and se transporter constructs that were used earlier to transform CEN.PK2-1D. However, the seAGT1 construct could not be successfully transformed to any of these three yeasts. The reason for this is not known but possibly overexpression of se*AGT1* from the multicopy plasmid caused non-bearable effects for these yeasts, as no viable transformants were found. Results were therefore obtained only for scAGT1, and the se- and sc-type MALx1 constructs.

**Figure 4.**
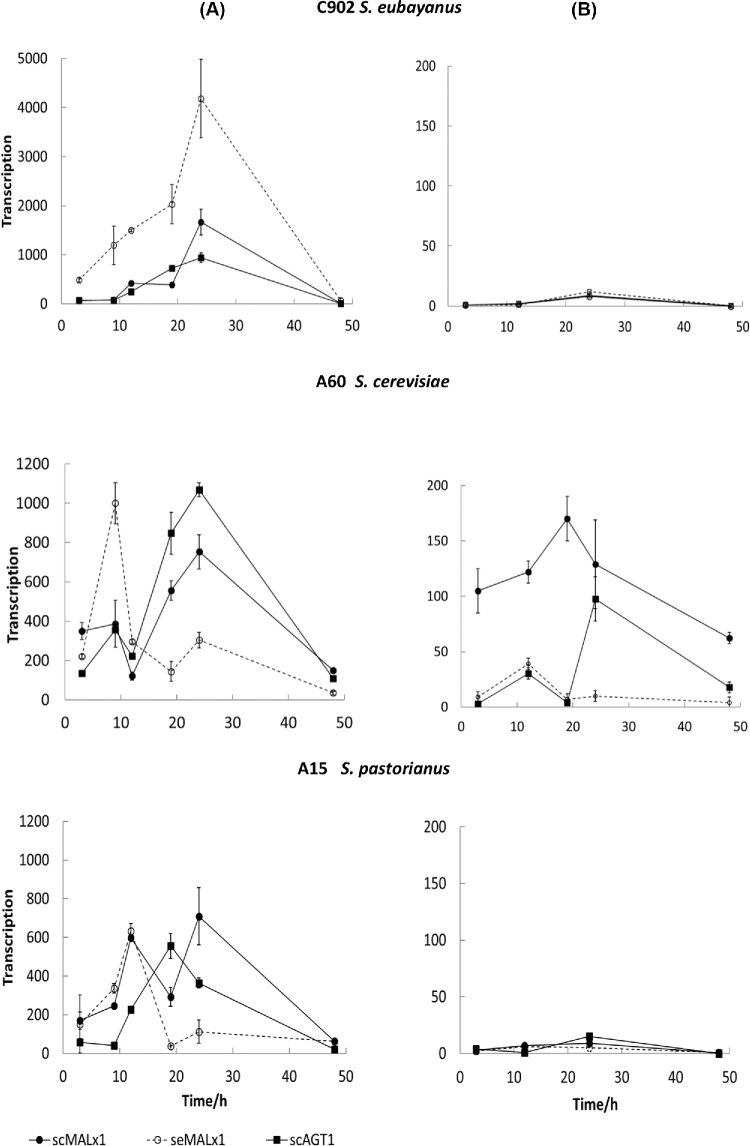
Gene transcription in *S. eubayanus* C902, *S. cerevisiae* A60 and *S. pastorianus* A15 hosts transformed with sc- and se-type transporters and grown on **(A)** maltose and **(B)** glucose. Results are averages ± ranges of data from two replicate growths of each yeast strain on each sugar. Due to great difference in transcription level between maltose and glucose ordinate scales are different.

All maltose transporter genes studied, both se- and sc-type, were induced strongly by maltose in all hosts (Fig. 4A), showing clearly that transregulation across *S. eubayanus* and *S. cerevisiae* genomes occurs for *MAL* genes. However, in the C902 host its ‘own gene’ se*MALx1*, i.e. gene originally cloned from its own genome, had higher transcription levels than the sc-type genes. Reciprocally, in the *S. cerevisiae* host, transcription of sc-type genes was more efficient compared to the se-type gene during most of the growth period. A similar response was seen with the lager yeast hybrid. It seems that trans-activation was less efficient compared to cis-activation.

Timing of *MAL* gene transcription was also different between hosts. In the C902 host, transcription of all three genes followed a similar pattern, reaching their peak at the 24 h time point. However, for A60 and A15 hosts, timing between transcription of sc- and se-type transporters varied. In the A60 host se*MALx1* transcription peaked early in the growth, at the 10–12 h time point, whereas sc-type genes peaked only at 20–24 h time point. In the A15 host both *MALx1* genes peaked early, both at the 12 h time point. In addition, sc*MALx1* peaked again at the 24 h time point but its se-version did not show this second peak. In the A15 host, sc*AGT1* peaked at the 20 h time point. It seems that the transcription pattern in the A15 host (hybrid) was different to patterns seen in the C902 and A60 hosts and timing of transcription in the hybrid is not directly inherited from either of the parental species, *S. eubayanus* or *S. cerevisiae*, but formed its own distinct pattern. This seems reasonable as there is a mix of sc- and se-type MAL activators and Mig1 repressors that generate a hybrid-specific expression pattern, which does not directly follow that of the parents. All maltose transporter genes studied, with the possible exception of sc*MALx1* in the A60 host, were repressed in the presence of glucose (Fig. 4B). In the C902 and A15 hosts, glucose repression was strong and even after glucose exhaustion only very low transcription was observed. In the A60 host there was a basal level of sc*MALx1* transcription from the beginning of the growth when glucose concentration was high (20 g l^−1^) and expression remained at this constant basal level during the linear growth phase. Contrarily, the sc*AGT1* gene in the same host was apparently subject to glucose repression and weak expression was observed only after glucose had been exhausted, at around the 15 h time point. The third gene, se*MALx1*, was strictly under glucose repression regardless of host strain.

In the present study, the multicopy plasmid YEplac195 was used. For this there are multiple promoter regions present in the cell and there was some risk that transcription factors, Mig1 repressors and MAL activators, would be titered out within the cell. Results obtained showed that at least Mig1 factors were not titered out (Fig. 4). In the C902 and A15 hosts, glucose repression of all genes investigated was very strong during the whole growth period. This means that there were enough Mig1 factors to bind all promoters present. In the A60 and CEN.PK2 hosts (Figs. 4 and 2) grown on glucose there was a low level of expression of sc-type genes but not those of se-type. Mig1 factors were not titered out in these hosts either, as otherwise continuous repression of se-type genes would not be possible. The YEplac195 plasmid number is expected to be similar in the same host independent of the transporter it overexpresses. This means that there would have been enough Mig1 repressors also for sc-type genes overexpressed in A60 and CEN.PK2 hosts but for some other reason repression was alleviated. If MAL activators were titered out, it would lead to a situation where maximum expression peaks would have been higher, but it would not otherwise affect to expression pattern seen as Mig1 repressors were not titered out. In the present study, the maximum level of expression was limited to what can be obtained with native MAL activators present in the cell.

**Figure 5.**
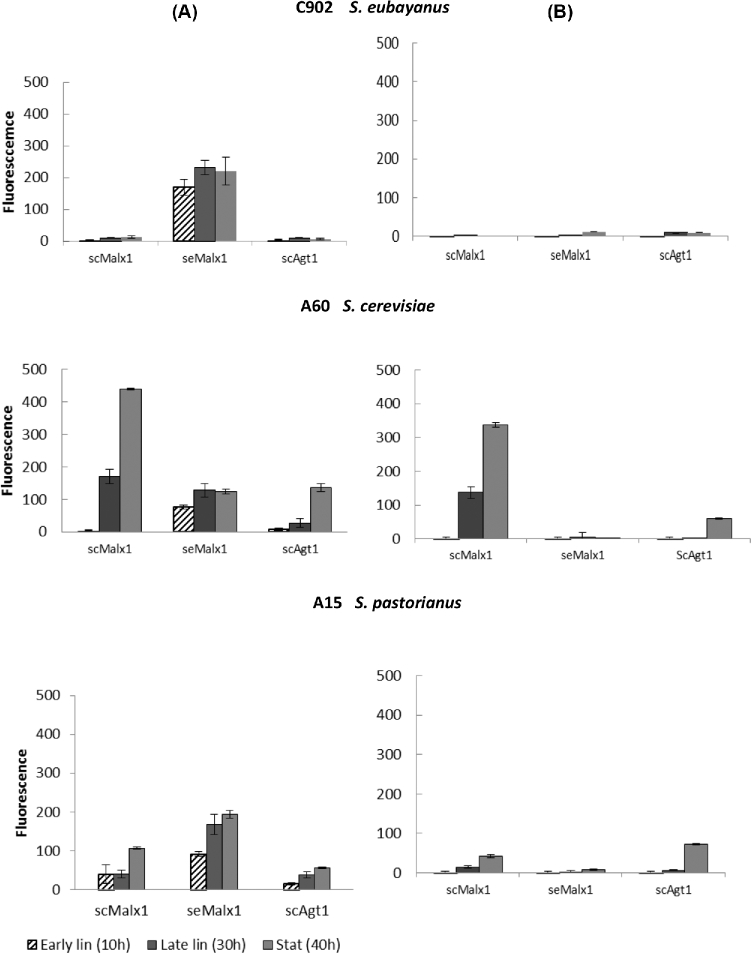
Amount of fluorescent signal in *S. eubayanus* C902, *S. cerevisiae* A60 and *S. pastorianus* A15 hosts transformed with sc and se-type transporters and grown on **(A)** maltose and **(B)** glucose. Samples were collected at early logarithmic growth phase at 10 h, at late logarithmic growth phase at 30 h and at stationary growth phase at 40 h. Results are averages ± ranges of data from two replicate growths of each yeast strain on each sugar.

The amount of fluorescence from each transporter, as determined by confocal microscopy, showed that, despite strong expression of foreign sc-type transporters in the C902 host, there were only low amounts of fluorescence for the actual transporters (Fig. 5A). This would suggest that sc-type transporter mRNAs detected by RT-qPCR were not translated efficiently to protein products, or these proteins were rapidly removed in *S. eubayanus*. Conversely, fluorescence form the native transporter of C902, seMalx1, was present in relatively high amounts during different growth phases of C902. This was consistent with strong expression of the gene. In the A60 and A15 hosts an early transcriptional peak for se*MALx1* was, apparently, sufficient to produce seMalx1 transporters for the whole period of growth as the amount of fluorescence for seMalx1 transporters remained quite stable during growth. For all strains, late transcription of sc-type genes correlated with an increasing fluorescence amount towards the stationary phase. In C902 and A15, glucose repression was strong and even after glucose exhaustion there was only a low transcription level observed. However, even this moderate transcription was enough to produce a small amount of transporter signals in cells (Fig. 5B). In the A60 host, even a low basal level of expression was enough to produce a significant amount of fluorescence for transporter protein.

**Figure 6.**
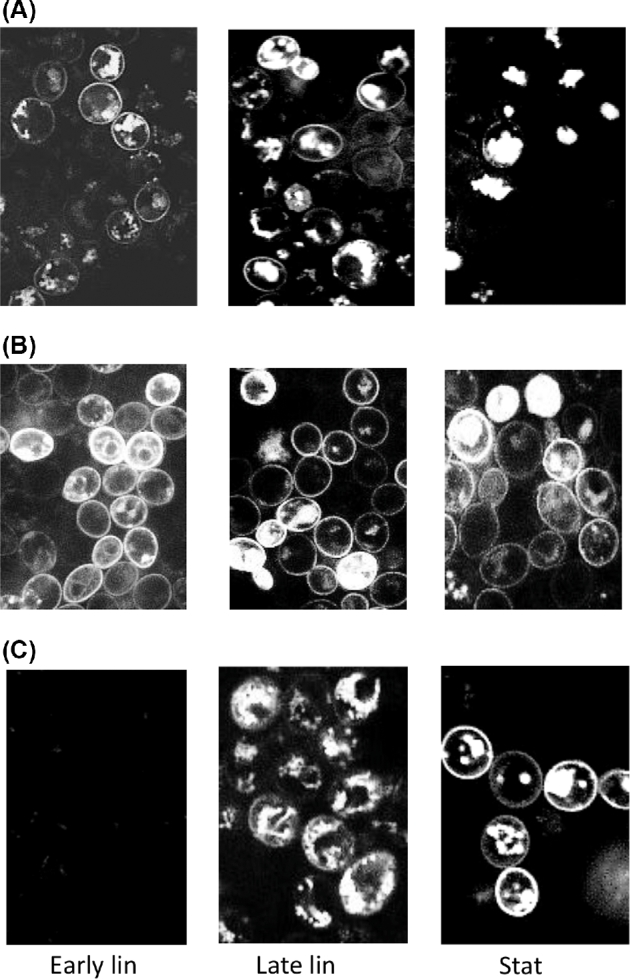
Fluorescent signals in A15 host for **(A)** scMalx1-mCherry, **(B)** seMalx1-Gfp and **(C)** scAgt1-mCherry in early linear, late linear and stationary growth phases on glucose, respectively.

Confocal microscopic studies showed that there was temporal variation in the localization of transporters on the plasma membrane depending on the transporter type. As an example, Fig. 6 shows localization of different transporters between plasma membrane and intracellular compartments in the A15 host. In Table 8 division of fluorescence between plasma membrane and intracellular compartments, determined by visual analysis of fluorescent images, in different hosts is shown. The seMalx1 transporter fluorescence was the most stable as it was found on the plasma membrane at a constant level during all growth phases in all hosts. In hosts where scAgt1 and scMalx1 transporters were present they were observed to have a certain pattern of localization on the plasma membrane, i.e. in both A15 and A60 hosts, signals for scMalx1 transporters were present in the plasma membrane during the linear growth phase, but were missing in the plasma membrane in the stationary growth phase. Possibly, they were already recycled back to intracellular compartments, as there were high amounts of fluorescence present in the intracellular space. Unfortunately, distinguishing between vacuoles and other intracellular compartments was not possible to do reliably based on data available. In the yeast cell the vacuole is a dynamic structure that can rapidly modify its morphology depending on needs of the cell and cell cycle stage and, as such, it does not have any basic size or shape. There can also be more than one vacuole per yeast cell. Staining of vacuoles with an organelle specific dye would have been needed to make this kind of distinction between vacuoles and other intracellular compartments. In contrast, signals for scAgt1 transporters were not found in the plasma membrane until stationary phase, even if they were abundant in intracellular compartments earlier. There seems, therefore, to be a mechanism excluding scAgt1 transporters from the plasma membrane until later phases of growth. In the C902 host, signals for seMalx1 transporters were present in the plasma membrane in high amounts during all growth phases, whereas very faint signals of sc-type transporters were found only in intracellular compartments. When yeast was grown on glucose, signals started to emerge in some of the hosts only in the late linear growth phase (Table 8). Also under repressive conditions signals for scMalx1 transporters appeared earlier in the plasma membrane.

**Table 8.** Localization of tagged transporters when grown on maltose and glucose in rich media. Localization determined at early linear phase (OD_600_ 2-4), late linear phase (OD_600_ 15-20) and stationary phase (after 40 h cultivation; OD_600_ >20). Determined from fluorescent images by visual inspection.

On maltose		On plasma membrane			In intracellular compartments		
		Early lin	Late lin	Stat	Early lin	Late lin	Stat
C902	ScMalx1	−	−	−	+	+	+
	SeMalx1	++	++	++	+	+	+
	ScAgt1	−	−	−	−	+	+
A60	ScMalx1	−	++	−	++	++	++
	SeMalx1	++	++	++	−	−	−
	ScAgt1	−	−	++	++	++	++
A15	ScMalx1	+	+	−	++	++	++
	SeMalx1	++	++	++	+	+	+
	ScAgt1	−	−	++	-	++	++
		Early lin	Late lin	Stat	Early lin	Late lin	Stat
C902	ScMalx1	−	−	−	−	−	−
	SeMalx1	−	+	+	−	+	+
	ScAgt1	−	−	−	−	−	−
A60	ScMalx1	−	+	+	−	+	+
	SeMalx1	−	−	−	−	−	−
	ScAgt1	−	−	+	−	+	+
A15	ScMalx1	−	+	+	−	+	+
	SeMalx1	−	−	−	−	−	−
	ScAgt1	−	−	+	−	+	+

^−^ Not visible.

^+^ Fluorescent signals.

^++^ Strong fluorescent signals.

When grown on glucose, no significant effect was seen in growth, ethanol production and glucose utilization in strains transformed with constructs (Fig. S4, Supporting Information), whereas on maltose differences were observed. In the A15 host, both se- and sc-type maltose transporter expression had beneficial effects, as a slightly higher ethanol production compared to the control strain was observed (Fig. S5, Supporting Information). In the A60 host, however, expression of seMalx1 did not have an effect compared to the control but both sc-type transporters, scAgt1 and scMalx1, decreased growth rate and ethanol utilization. In the C902 host, utilization of maltose as well as growth and ethanol production was delayed in all strains possessing the construct compared to host. This was especially apparent with the sc*AGT1* construct, where it was seen that maltose was not consumed efficiently and only approximately 2 g l^−^^1^ ethanol was produced.

## DISCUSSION

Results of the present study showed for the first time that MAL activators work beyond genome boundaries. Newly introduced sc- and se-type transporter genes were transcribed at a high level in both *S. cerevisiae* and *S*. *eubayanus* backgrounds, thus demonstrating trans-regulation across subgenomes. Sequence analysis showed low sequence conservation in the Mig1 and MAL activator binding sites described by Levine, Tanouye and Michels (1992), Bell *et al.* (1997) and Vidgren *et al.* (2011) between sc and se-type promoters. This was observed especially for *AGT1* promoters. However, CG-rich motifs forming binding sites for MAL activators described in Pougach *et al.* (2014) were found in all promoters of the present study. These CGC(9N)CGN and CGG(9N)CGG binding sites were most probably responsible for much of the *MAL* gene activation observed. Response to maltose required the presence of more than one MAL activator binding site and an increase in the number of such sites in the promoter from two to three increases the expression significantly (Pougach *et al.*^2014^). All other promoters in this study contained a sufficient number of CGC(9N)CGN and CGG(9N)CGG binding sites (two) for expression. The exception were the sc-type *AGT1* promoters, which contained only one site. Since sc-type *AGT1* promoters possessed other types of MAL activator binding sites (Vidgren *et al.*^2011^), activation might have occurred in combination with other MAL activator binding elements and this one CGC(9N)CGN binding site. It is possible that also in other promoters, where other MAL activator binding sites are present, they intensify the effect conferred with two CGC(9N)CGN and CGG(9N)CGG binding sites. However, sc- and se-type activators were not completely interchangeable, as stronger activation of the gene was observed in the native host with native MAL activators. Also, timing of transcription varied depending on activator type. While the se-type MAL activator induced both orthologous genes simultaneously, the sc-type activator caused different timing in transcription, so that se-type genes were induced earlier than sc-type genes. These results are similar to those of an earlier investigation on sc- and se-type *ARO10* (2-oxo-acid decarboxylase) genes, where it was shown that trans-regulation across subgenomes occurs in lager yeast but, correspondingly, that se- and sc-type activators were not wholly interchangeable, as a basal level of se-type *ARO10* gene expression required the se-type activator, even if under inducing conditions both activator types were similarly efficient for both orthologous genes (Bolat *et al.*^2013^).

Comparison of transcription patterns in different hosts showed that in the lager hybrid the pattern was not directly inherited from either of the parents as such but instead was a combination of characteristics derived from each parent, i.e. strong glucose repression of *MAL* genes was similar to that found in *S. eubayanus*, whereas the transcription pattern of *MAL* genes under inducing conditions was more similar to that seen in the *S. cerevisiae* parent. The observation that the *MAL* gene activation pattern followed that of *S. cerevisiae* suggests that MAL activators which are operational in this context are mostly of the sc-type. This seems reasonable as there are more sc-type *MAL* loci present in the lager hybrid (Nakao *et al.*^2009^) and it is expected that there are more sc-type activators present relative to se-type. Hybrid-specific gene expression has been found in other studies also. Tirosh *et al.* (2009) performed allele-specific expression profiling of two yeast species, *S. cerevisiae* and *S. paradoxus*, and an artificially made hybrid of these two. For many genes they observed hybrid-specific expression deviating from the parental pattern. They explained most of these changes by novel cis-trans interactions or through modified trans-regulation associated with environmental sensing and signalling.

In general, it seems to be common that, once the hybrid has formed, the transcription pattern of orthologous genes is co-regulated. This means that timing of transcription for orthologous genes follows the same pattern (Horinouchi *et al.*^2010^). This seems reasonable as promoters of orthologous genes possess similar activator and repressor binding elements and are exposed to the same mixture of cis- and trans-factors in the hybrid cell. However, even if the timing of transcription followed the same pattern, differences in the level of gene transcription between orthologous genes have been found (He *et al.*^2014^; Gibson *et al.*^2015^). Nevertheless, in the present study sc- and se-type *MAL* genes were not found to be co-regulated. Early induction of se-type *MAL* genes compared to sc-type genes was observed in all other hosts except C902. Studies by Gibson *et al.* (2013) had already shown some examples where in the lager hybrid there are differences in timing of transcription of orthologous maltose metabolism genes and possibly this is the characteristic of *MAL* genes.

In the C902 host, high transcription levels of both sc-type transporter genes did not correlate with transporter amount detected in cells. It seems that the sc-type transporter mRNAs are not translated to mature, folded transporter proteins efficiently or, if they are, they are unstable and degraded rapidly. Nevertheless, a small amount of both scAgt1 and scMalx1 transporter proteins present in C902 cells showed that protein translation is not totally defective. Poor conversion of mRNA to sc-type transporters could be due to, for example, a missing lipid component in C902 membranes. As transporters are in effect protein-lipid complexes, they need certain lipids for formation of a functional structure. Lipid composition is important for determining the correct topological organization of transporters, either during the initial membrane assembly or dynamically after membrane insertion has occurred (Bogdanov, Heacock and Dowhan 2002). In general, in cold tolerant strains, such as *S. eubayanus*, lipid composition of cells differs from non-cold tolerant strains (Krogerus *et al.*^2017^). Changes in the lipid composition may be an adaptive response to low temperature fermentations (Torija *et al.*^2003^). A study with the Fur4 transporter has shown that conformational alterations in the transporter caused its rapid degradation. When different versions of the Fur4 transporter tagged with Gfp were studied with fluorescence microscopy, it was observed that versions with non-folding mutations were barely detectable by fluorescence microscopy because of rapid degradation (Keener and Babst 2013). Fluorescent images in the present study concerning sc-type transporters in the C902 host were remarkably similar to those obtained with Fur4 non-folding mutants.

In the lager hybrid, one of the two orthologous genes encoding α-glucoside transporters is mutated and non-functional (Vidgren, Ruohonen and Londesborough 2005). It has been suggested that orthologous transporters compete with each other for limited resources of delivery and stable localization of transporters into the plasma membrane. It can be hypothesized that the se-type Malx1 transporters, which are constantly present in the plasma membrane, limit membrane space for other transporters. During the fermentation process, wide-substrate-range transporters (especially for maltotriose) are important, and their presence in the plasma membrane is needed for efficient fermentation. The presence of se-type Malx1 transporters may not therefore be suitable for brewing yeast, which must contend with a dynamic substrate composition during fermentation. Consequently, loss-of-function mutations in the se*MALx1* gene may actually have been beneficial for lager yeast in its typical environment and this may explain why only scMalx1 transporters are functional in present-day lager yeast.

As for *MALx1*, in the case of the *AGT1* orthologous gene pair in lager yeast, one of the transporters (sc-type) is mutated and non-functional (Vidgren and Londesborough 2012; Cousseau *et al.*^2013^). However, both sc- and se-type Agt1 transporters seem to localize to the plasma membrane similarly at late stages of growth. Also, they are both maltose/maltotriose transporters having the same wide substrate range (Vidgren *et al.*^2010^). A possible reason for seAgt1 being more suitable in the hybrid background is that it functions better at the low temperatures typical of lager fermentations. The seAgt1 transporters retain more maltose and maltotriose uptake activity at low temperatures compared to scAgt1, especially below 10°C which is a temperature traditionally employed in lager fermentations (Vidgren *et al.*^2010^).

Thus, it seems that lager yeasts may have adapted to the brewing environment by accumulating loss-of-function mutations in genes that were less suited to brewing conditions. Also, these yeast appear to be capable of fine-tuning sugar uptake during fermentation by changing the main transporters present in the plasma membrane in response to changing substrate composition. Firstly, maltose, the most abundant sugar in the wort, is taken up by scMalx1 transporters, which are highly active and specific for maltose. In later phases of growth, scMalx1 transporters are withdrawn and seAgt1 transporters, which have a wider substrate range and operate well at low temperatures, are carried to the plasma membrane so that remaining sugars of wort, and in particular maltotriose, can be taken up by the cells.

In conclusion, with the *MALx1* orthologous gene pair, differential timing of localization of the respective transporters to the plasma membrane feasibly confers a competitive advantage upon the scMalx1 transporter. With the *AGT1* orthologous pair, the competitive advantage may be the greater suitability of the seAgt1 type transporter to low-temperature fermentations. Both conjectures could be tested by evolving de novo hybrid yeast in brewing-relevant conditions and determining the rate at which loss-of-function mutations arise in the respective transporter genes.

## SUPPLEMENTARY DATA

Supplementary data are available at *FEMSYR* online.

Supplemental Figures
