# Supplementary material for: Trans-regulation and localization of orthologous maltose transporters in the interspecies lager yeast hybrid
Source: FEMS Yeast Res. 2018 Jun 19;18(6):foy065. doi: 10.1093/femsyr/foy065 (PMC6142294; doi:10.1093/femsyr/foy065)
Supplement: Supplemental Figures [file foy065_supplemental_files.zip › Supplementry_caption.docx]

**Fig S1.** Mig1 and MAL activator binding sites in divergent *MALx1-MALx2* promoter (Levine *et al*.1992, Bell *et al*. 1997) compared to sc and se-type *MALx1* promoters of present study. Binding sites CGC(9N)CGN and CGG(9N)CGG for MAL activator binding reported by Pougach *et al*. 2014 are included. Sequence for sc*MALx1* promoter is from sequenced lager strain WS34/70 in sc-type Chr II contig208.1 (Nakao *et al*. 2009); Sequence for se*MALx1* promoter is from sequenced *S. eubayanus* type strain CBS12357^T^ in Chr XVI (Libkind *et* *al*. 2011). Sequences start -1 relative to the start codon of *MALx1* genes. Multiple sequence alignment with hierarchical clustering (Corpet, 1988).

**Fig S2.** Mig1 and MAL activator binding sites in sc and se-type *AGT1* promoters of present study. Sequence for sc*AGT1* promoter used at present study was from A60 ale yeast and has been sequenced in Vidgren *et al*. 2011. Computational promoter element analysis has been performed in Vidgren *et al*. 2011 for A60 *AGT1* promoter and corresponding promoter in A15 lager yeast. Promoter elements found in Vidgren *et al*. 2011 are shown in figure. Sequence for se*AGT1* promoter is from A15 lager yeast and sequence is identical to that of sequenced lager yeast WS34/70 se-type ChrII (Nakao *et al*. 2009). Binding sites CGC(9N)CGN and CGG(9N)CGG for MAL activator binding reported by Pougach *et al*. 2014 are included. Sequences start -1 relative to the start codon of *AGT1* genes. Multiple sequence alignment with hierarchical clustering (Corpet, 1988).

**Fig S3**. Growth, ethanol and sugar concentrations in cultivations performed with CEN.PK2-1D host transformed with sc and se-type transporters and grown on A) maltose and B) glucose. Results are averages ± ranges of data from two replicate growths of each yeast strain on each sugar.

**Fig S4.** Growth, ethanol and sugar concentrations in cultivations performed with *S. eubayanus* C902, *S. cerevisiae* A60 and *S. pastorianus* A15 hosts transformed with sc and se-type transporters and grown on glucose. Results are averages ± ranges of data from two replicate growths of each yeast strain.

**Fig S5**. Growth, ethanol and sugar concentrations in cultivations performed with *S. eubayanus* C902, S. cerevisiae A60 and *S. pastorianus* A15 hosts transformed with sc and sc-type transporters and grown on maltose. Results are averages ± ranges of data from two replicate growths of each yeast strain.
