# Supplementary figures and images for: Trans-regulation and localization of orthologous maltose transporters in the interspecies lager yeast hybrid
Source: FEMS Yeast Res. 2018 Jun 19;18(6):foy065. doi: 10.1093/femsyr/foy065 (PMC6142294; doi:10.1093/femsyr/foy065)

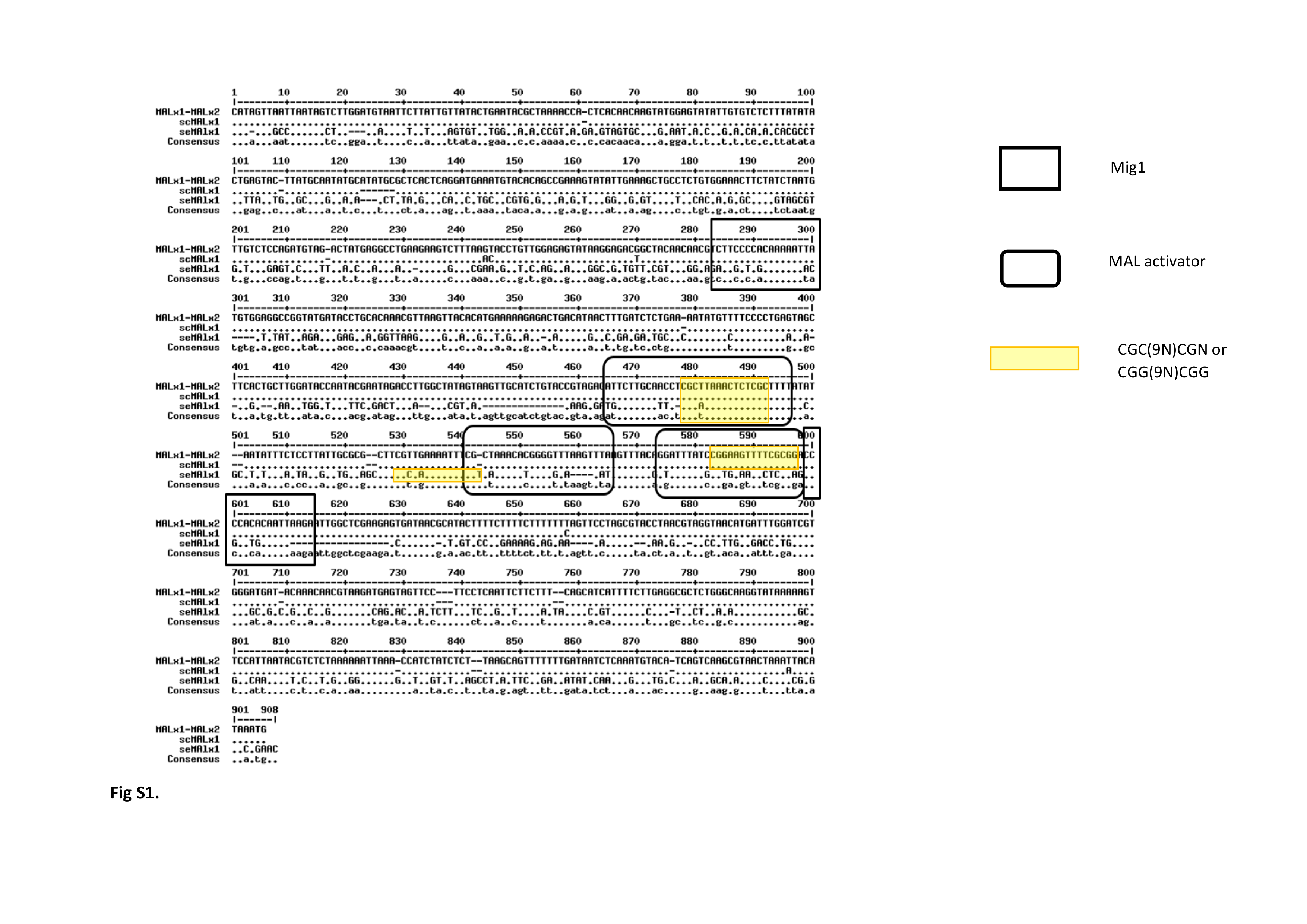

Supplement: Supplemental Figures [file foy065_supplemental_files.zip › FigS1.tif]

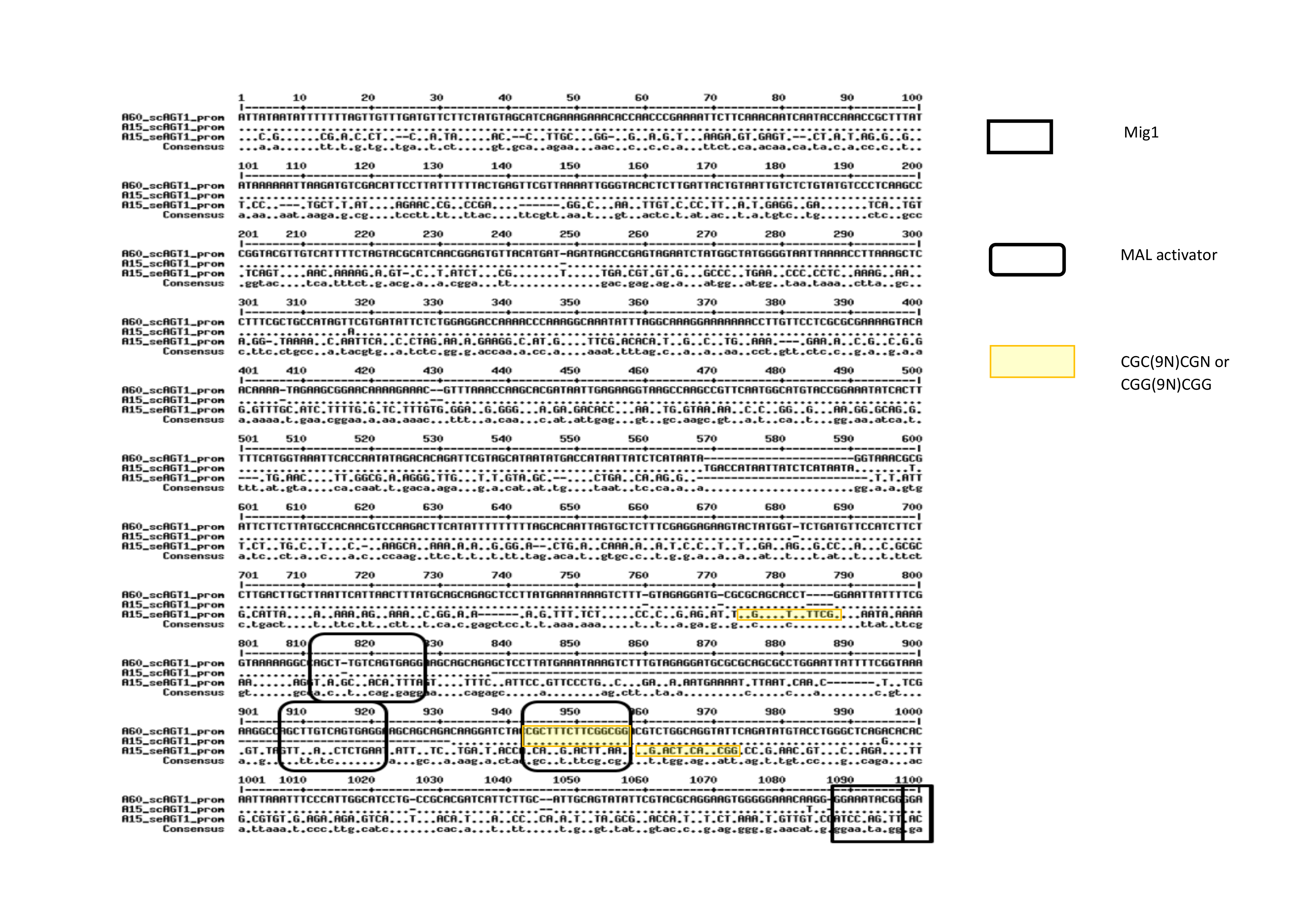

Supplement: Supplemental Figures [file foy065_supplemental_files.zip › FigS2.tif]

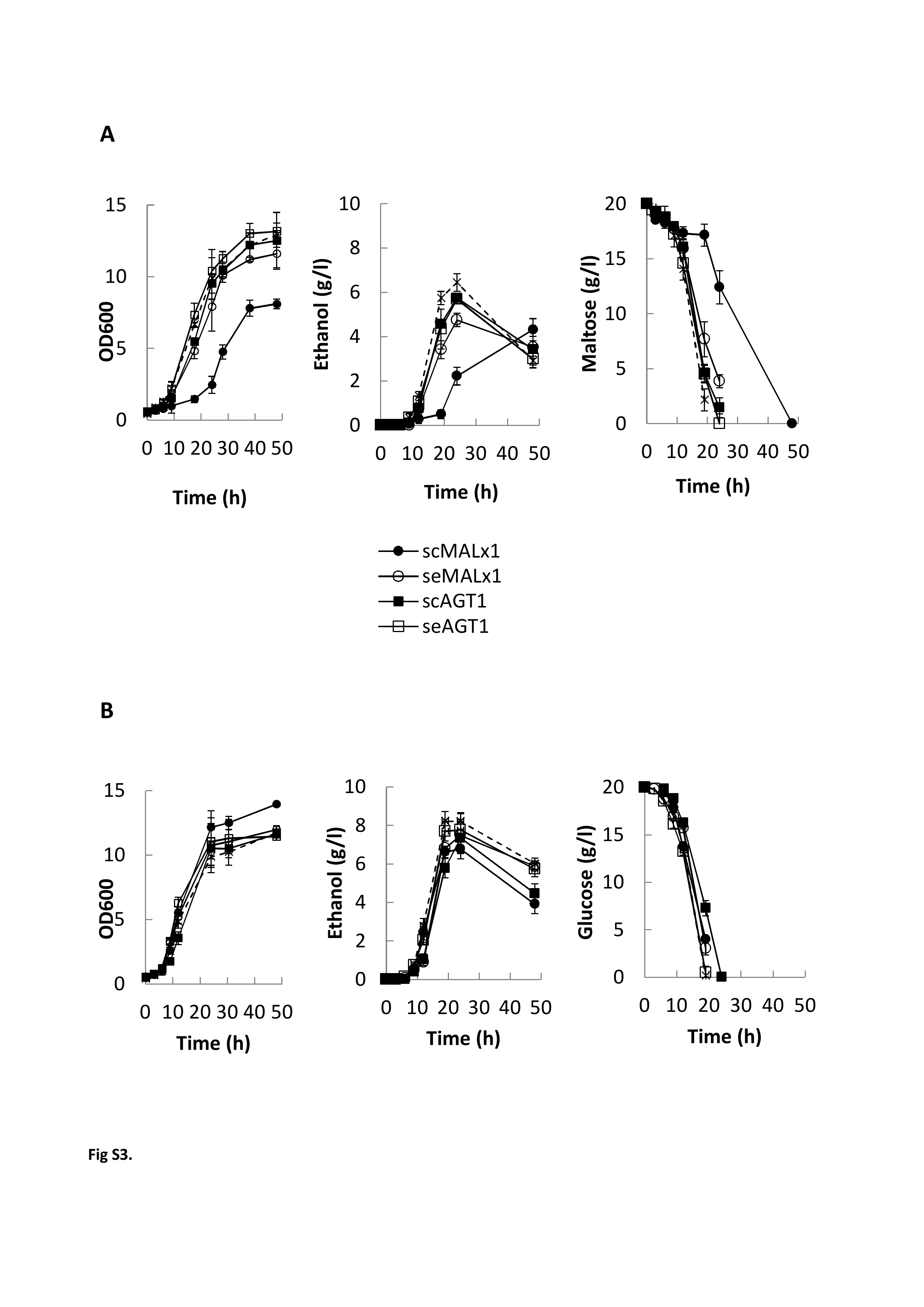

Supplement: Supplemental Figures [file foy065_supplemental_files.zip › FigS3.tif]

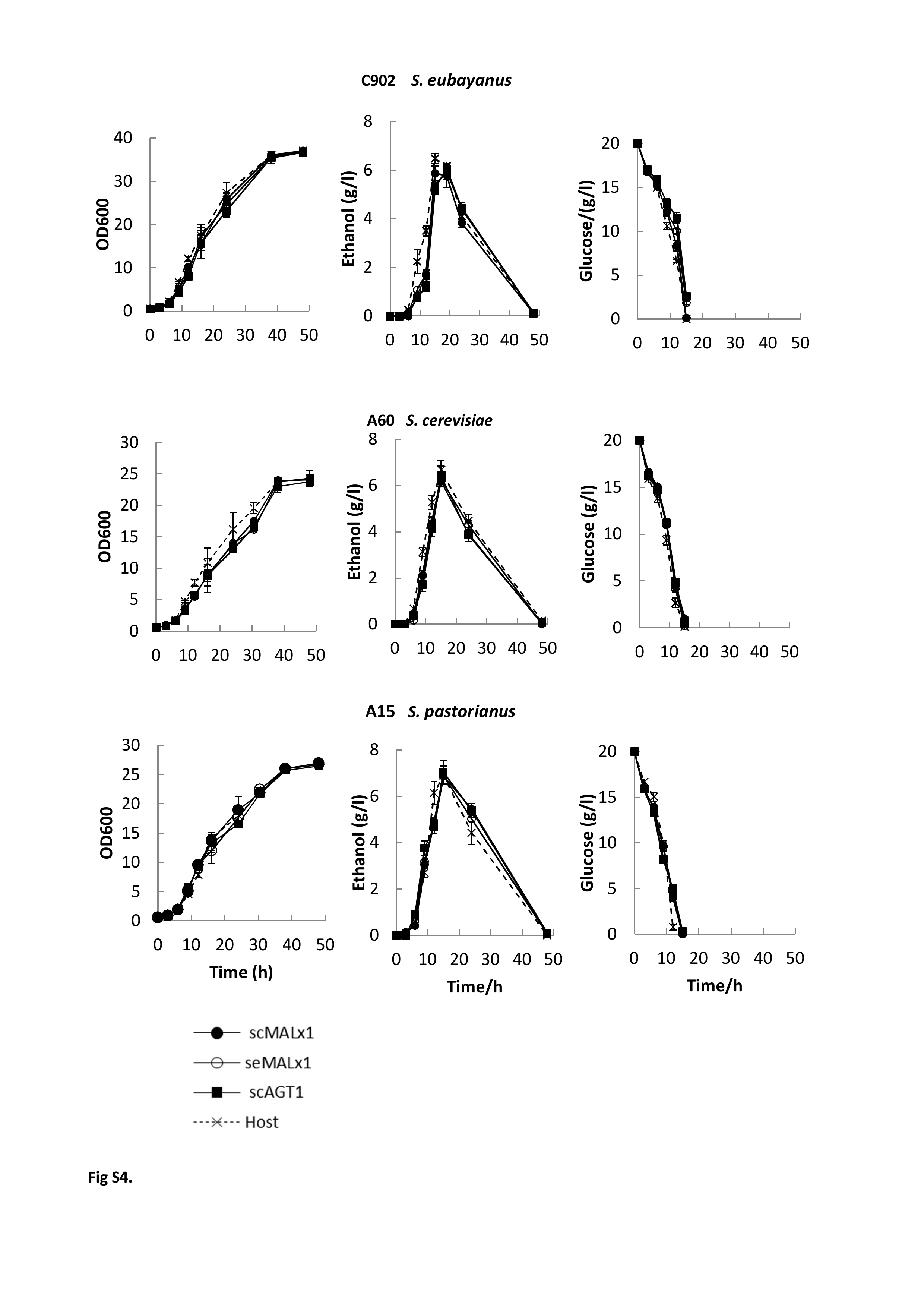

Supplement: Supplemental Figures [file foy065_supplemental_files.zip › FigS4.tif]

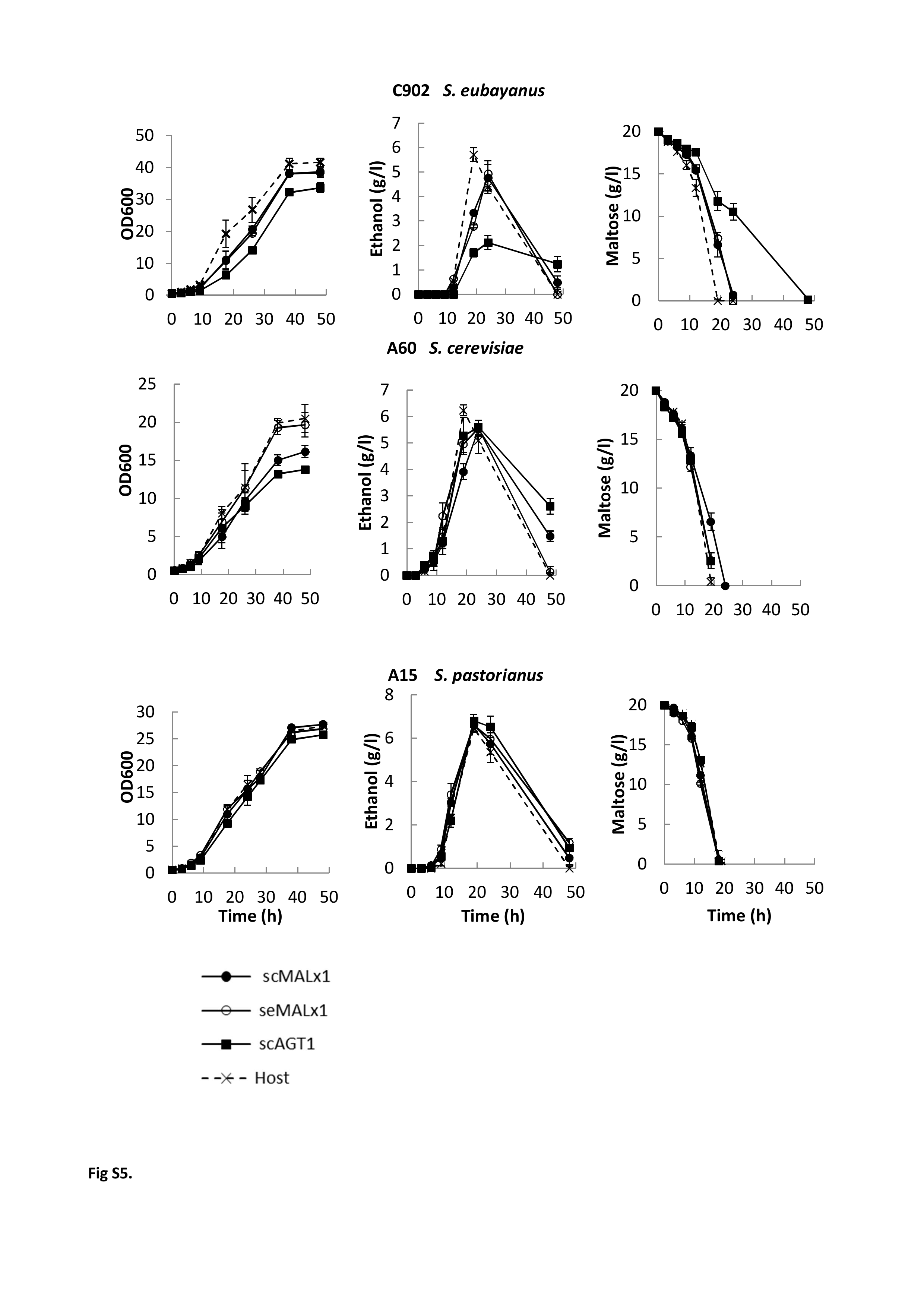

Supplement: Supplemental Figures [file foy065_supplemental_files.zip › FigS5.tif]
